# In-situ γ-ray analysis of ground surface radioactivity using portable HPGe γ spectrometer

**DOI:** 10.1038/s41598-022-13770-5

**Published:** 2022-06-08

**Authors:** Zeqian Wu, Bairong Wang, Jian Sun, Yuqi Wang, Changwei Zhao

**Affiliations:** 1 Institute of NBC Defence, Changping District, Beijing, 102205 China; 2 College of Resources and Environmental Sciences, China Agricultural University, Haidian District, Beijing, 100193 China

**Keywords:** Experimental nuclear physics, Experimental particle physics, Nuclear waste

## Abstract

As essential high-end equipment for nuclear emergency monitoring, the portable HPGe γ spectrometer currently lacks supporting in-situ measurement methods, limiting its role and value in emergency missions. For this practical problem, this paper studies the measurement of ground surface radioactivity by portable HPGe γ spectrometer in nuclear emergency monitoring in view of the particularity of nuclear emergency source items. Firstly, the detection efficiency of point sources at different horizontal distances when the spectrometer is installed at the height of 1 m from the center of the detector to the ground is calculated. Secondly, the concept of effective contribution distance is defined and analyzed. Thirdly, the point source detection efficiency is obtained using the numerical integration method of calculation. Integrate to calculate the detection efficiency of the surface source, and then calculate the radioactive surface activity of the surface. Finally, the effectiveness of the method is verified through experiments.

## Introduction

With the development of science and technology, nuclear energy and nuclear technology have gradually embarked on a large-scale platform for human life. As nuclear power plants are widely used, they also bring some risks. As the last barrier to protect public safety, a nuclear emergency is worthy of attention and related technical research. Emergency radiation monitoring is an integral part of a nuclear accident emergency. The International Atomic Energy Agency (IAEA) proposes that one of nuclear accident emergency monitoring purposes is to provide accurate and timely information to the degree of radiation hazards^1^. The International Commission on Radiological Protection (ICRP), IAEA, and other organizations^2–4^ have put forward the concept of Operational Intervention Level (OIL) to make correct decisions in response to nuclear reactor accidents. An effective γ-ray radioactivity monitoring method is used to compute these values.

There are two main methods for measuring surface activity after a nuclear accident: laboratory analysis and in-situ measurement. The former focuses on on-site sampling and returns to the laboratory for more accurate analysis. At the same time, in-situ measurement refers to the collection and processing of data on the spot and directly gives the value of the physical measurement concerned, which has the advantages of convenience and speed.

At present, the primary method used for in-situ measurement is the Beck method, but the calibration process of the Beck method is complicated and time-consuming^5^. Some subsequent studies have optimized the in-situ measurement methods^6–19^, including a lot of work applied to geological surveys, some applied to accident emergency conditions, and some studies on in-situ measurements on the seabed. The radioactivity of the monitored objects is low in geological surveys, and the depth distribution of radionuclides has an essential impact on the measurement results. For in-situ measurements in seawater, there is a large difference in the attenuation coefficient of the medium for γ-rays, which will affect the scaling process of detection efficiency. However, when applied to nuclear emergency monitoring, the radioactivity of the monitored object is relatively high. In the early stage of the nuclear accident, the radionuclides settled on the surface have not penetrated the ground.

So it is necessary to simplify and improve it according to the characteristics of different usage scenarios. The basic premise and scope of application of this method are that in the early stage of a nuclear accident, the radioactive pollution that settles on the ground surface can be regarded as a surface source. It can be considered that there is no radioactive material in the air. Based on this, an effective and convenient in-situ measurement method of surface activity is established.

## Methodology

**Figure 1:**
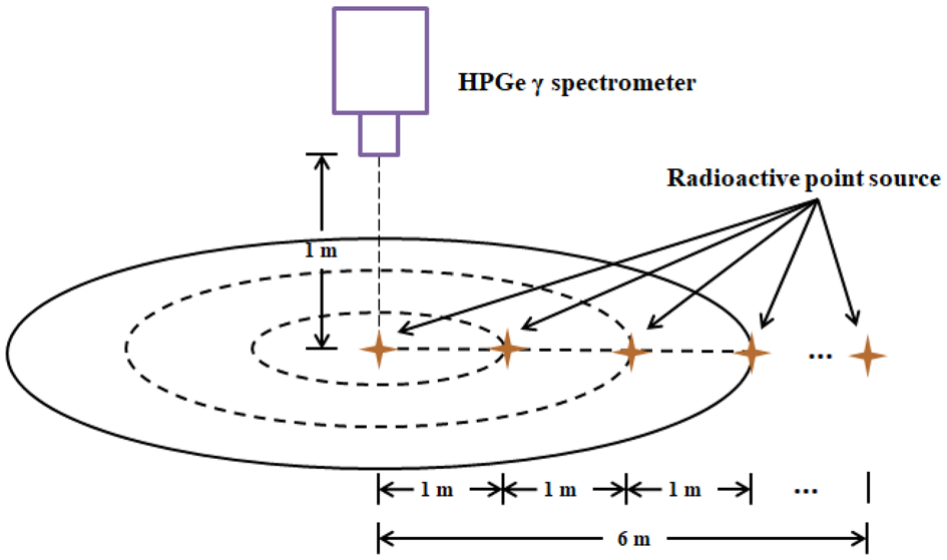
Schematic diagram of the detection model. The HPGe γ spectrometer was erected at a height of 1 m from the ground using a tripod, and the γ radiation source is placed at a horizontal distance of 0–6 m from the detector, and the interval is 1 m.

The HPGe γ spectrometer has different detection efficiency for γ-ray with different energies. Therefore, to obtain the detection efficiency of low-, medium-, and high-level energy γ-ray, experiments with different energy rays need to be performed. The Monte Carlo Simulation is adopted to simulate the point source detection efficiency at different energies and locations. The horizontal distance between the radiation source and the center of the detector extends from 0 to 6 m, as is shown in Fig. 1.

**Figure 2:**
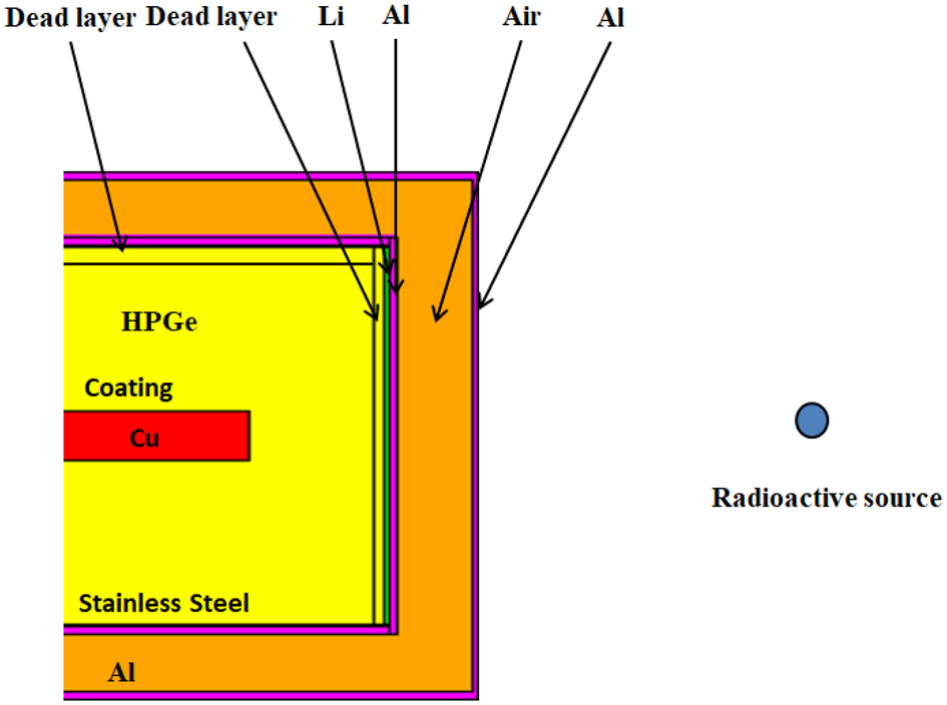
Structure of the characterized HPGe detector used for modeling in Monte Carlo simulations.

The Monte Carlo simulation relies on the accurate characterization of the structure of the portable HPGe γ spectrometer. Figure 2 describes the structure of the detector used in Monte Carlo simulation.

The calculated detection efficiency and relative uncertainty for different energy gamma rays at different positions are shown in Table 1.

**Table 1:** Variation of the detection efficiency of different energy γ-ray with horizontal distance.

Nuclide (energy/keV) (m)	^132^Te (228)	^131^I (364)	^133^I (530)	^137^Cs (662)	^60^Co (1173)	^60^Co (1332)	^124^Sb (1691)
0	1.56E−04 (0.57%)	1.07E−04 (0.68%)	7.80E−05 (0.80%)	6.50E−05 (0.88%)	4.10E−05 (1.11%)	3.70E−05 (1.16%)	3.00E−05 (1.28%)
1	7.55E−05 (0.81%)	5.13E−05 (0.99%)	3.70E−05 (1.16%)	3.08E−05 (1.27%)	1.98E−05 (1.59%)	1.77E−05 (1.68%)	1.50E−05 (1.83%)
2	2.79E−05 (1.34%)	1.92E−05 (1.61%)	1.41E−05 (1.88%)	1.16E−05 (2.08%)	7.73E−06 (2.54%)	7.12E−06 (2.65%)	5.82E−06 (2.93%)
3	1.29E−05 (1.97%)	8.92E−06 (2.37%)	6.54E−06 (2.76%)	5.43E−06 (3.04%)	3.82E−06 (3.62%)	3.54E−06 (3.76%)	2.86E−06 (4.18%)
4	7.38E−06 (2.60%)	5.24E−06 (3.09%)	3.69E−06 (3.68%)	3.25E−06 (3.93%)	2.13E−06 (4.85%)	1.93E−06 (5.10%)	1.56E−06 (5.67%)
5	4.63E−06 (3.29%)	3.24E−06 (3.93%)	2.32E−06 (4.65%)	2.02E−06 (4.98%)	1.40E−06 (5.99%)	1.27E−06 (6.29%)	1.15E−06 (6.59%)
6	3.10E−06 (4.02%)	2.29E−06 (4.68%)	1.60E−06 (5.59%)	1.34E−06 (6.11%)	9.10E−07 (7.41%)	8.50E−07 (7.67%)	7.10E−07 (8.39%)

Adopt the least square method to fit the above detection efficiency value according to Eq. (1),

1 $$ \varepsilon_{E} (r) = ae^{ - br} $$

where *ε*_*E*_(*r*) is the characteristic γ-ray with energy *E*, the point source detection efficiency at a horizontal distance of r meters; *r* represents the horizontal distance between the point source position and the center of the detector, m; *a* and *b* represents fitting coefficient. The calculation result is shown in Table 2.

**Table 2:** The parameter value of the fitting equation for detection efficiency.

Radionuclide	Energy (keV)	The parameter value of the fitting equation		Fitted regression coefficients *R*^2^
		*a*	*b*	
^132^Te	228	1.59E−04	8.50E−01	0.9957
^131^I	364	1.08E−04	8.25E−01	0.9972
^133^I	530	7.87E−05	8.13E−01	0.9981
^137^Cs	662	6.49E−05	7.95E−01	0.9968
^60^Co	1173	4.11E−05	7.83E−01	0.9980
^60^Co	1332	3.70E−05	7.67E−01	0.9955
^124^Sb	1691	3.05E−05	7.59E−01	0.9976

**Figure 3:**
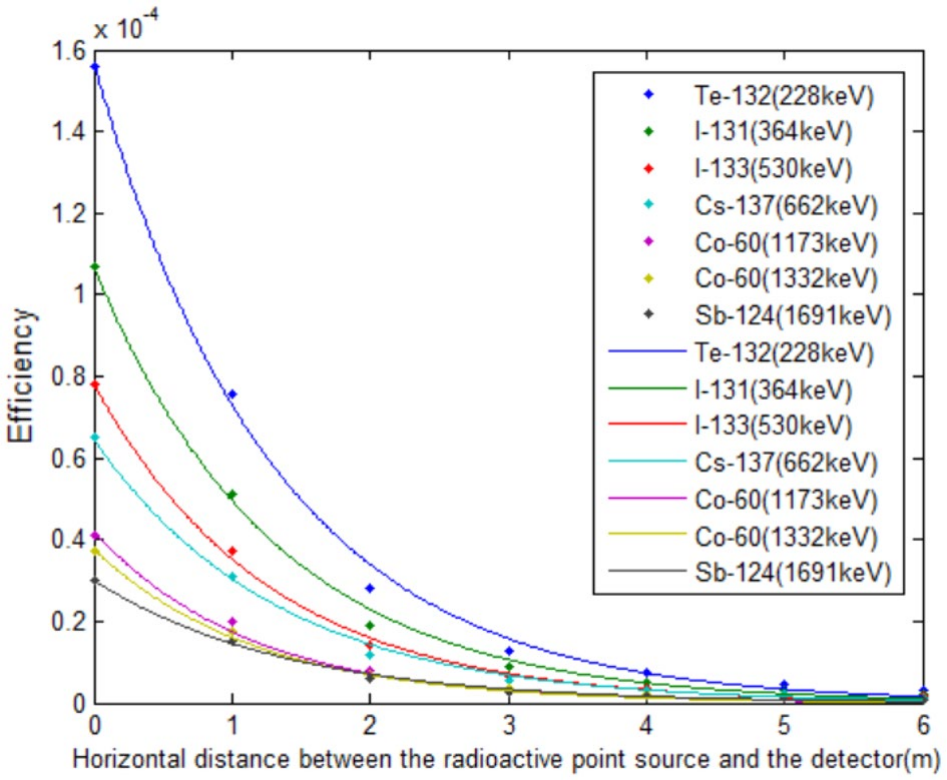
The HPGe γ spectrometer has different detection efficiency for characteristic γ-ray of different energy, and it decreases rapidly as the horizontal distance increases. The characteristic γ-ray of different energy is shown in different colors in the picture.

The detection efficiency curve is reverted to exponential form, and the fitting result is shown in Fig. 3.

It can be observed that the fitting effect is good. As the energy increases, the detection efficiency curve moves downwards overall, indicating that in this detection model, the higher the ray energy, the lower the detection efficiency of the point source at the exact location.

When the detector is placed in a flat and open area and 1 m away from the ground, its detection object can be regarded as a significant and uniformly distributed radioactive surface source. The practical contribution distance of HPGe γ spectrometer to γ-ray is of great significance for radioactivity measurement of significant surface sources.

The practical contribution distance of the surface source to the spectrometer can be determined by setting the threshold for the contribution of artificial radionuclides to the spectrometer. The contribution of the radioactive point source to the spectrometer can be expressed by the spectrometer’s counting rate of γ-ray, and the calculation equation of the counting rate is shown in Eq. (2),

2 $$ n = A \cdot p \cdot \varepsilon_{E} (r) \cdot K $$

where *n* represents the spectrometer’s counting rate of a certain γ-ray, cps; *A* represents the activity of the point source, Bq; *p* represents the branching ratio of the characteristic gamma-ray; *ε*_*E*_(*r*) represents detection efficiency of the point source at a horizontal distance *r*; *K* represents decay correction factor during measurement.

Because the measurement object is approximately a surface source and the spectrometer is set up at the height of 1 m from the ground, the effects of self-absorption correction and pulse coincidence correction can be ignored. However, due to the short half-life of some key radionuclides, the change of its activity needs to be taken into account in the actual measurement process. Therefore, the decay correction coefficient *K* during the measurement is introduced. The calculation equation is shown in Eq. (3) ^20^,

3 $$ K = \frac{{T_{1/2} }}{\ln 2 \times t}\left[ {1 - \exp \left( { - \frac{\ln 2 \times t}{{T_{1/2} }}} \right)} \right] $$

where *T*_1/2_ represents the half-life of the nuclide to be tested, s; *t* represents measurement time, s. The surface radioactivity after a nuclear accident can be regarded as a surface source, and it can be considered that there is no radioactive contamination in the air. Since the detection model is center symmetric, the contribution of the surface source to the spectrometer’s counting rate can be calculated by double integration based on the fitting equation of the point source detection efficiency with horizontal distance

4 $$ n_{s} = \iint\limits_{D} {A \cdot p \cdot \varepsilon_{E} (r) \cdot K}d\sigma = \frac{{2\pi a[1 - (br + 1)e^{ - br} ] \cdot A \cdot p \cdot K}}{{b^{2} }} $$

where *n*_*s*_ represents the contribution of the surface source to spectrometer counting rate, cps; *r* represents the radius of the surface source, m. The farther away the source is from the spectrometer, the smaller the contribution to the spectrometer’s count rate. The integration converges as the integration distance approaches infinity, which means that when the detection distance gradually increases to infinity, the contribution of the counting rate gradually approaches a specific value, that is, Eq. (5)

5 $$ n_{s\_\max } = \mathop {\lim }\limits_{r \to + \infty } A \cdot p \cdot K \cdot \int_{0}^{2\pi } {d\theta \int_{0}^{r} {ae^{ - br} rdr} } = A \cdot p \cdot K \cdot \frac{2\pi a}{{b^{2} }} $$

where *r* represents radius of the area source under consideration, m; *a*, *b* represents coefficients of the fitting equation. When the detection radius reaches a certain value, the contribution to the spectrometer’s count rate within this detection range will reach a part of the maximum contribution value. Calculate the horizontal distances at the 90% and 95% of the contribution of the maximum counting rate of the γ-ray of different energy, and establish the equation relationship,

6 $$ n_{s\_0.90} = 0.9n_{s\_\max } $$

7 $$ n_{s\_0.95} = 0.95n_{s\_\max } $$

where *n*_*s_*0.90_ represents 90% of the characteristic γ-ray’s contribution to the maximum counting rate, cps; *n*_*s_*0.95_ represents 95% of the characteristic γ-ray’s contribution to the maximum counting rate, cps. It is an integral equation. Calculate the detection distances at 90% and 95% of the maximum count rate contribution to γ-ray of different energy, as is shown in Table 3.

**Table 3:** Contribution distance to γ-ray of different energy.

Radionuclide	Energy (keV)	90% contribution distance (cm)	95% contribution distance (cm)
^132^Te	228	457.6	558.1
^131^I	364	471.5	575.0
^133^I	530	478.4	583.5
^137^Cs	662	489.3	596.7
^60^Co	1173	496.8	605.9
^60^Co	1332	507.1	618.5
^124^Sb	1691	512.5	625.0

Since the theoretical corresponding detection area is infinite when 100% of the maximum count rate is reached, this is unrealistic and cannot be calculated quantitatively. We usually consider the detector's detection capability and measurement time to be limited. For areas beyond a certain range, the contribution to the count rate of the detector is negligible. In this paper, the horizontal distance at 95% of the maximum count rate contribution of the spectrometer is defined as the effective contribution distance of the spectrometer, and the practical contribution distance is used to calculate the spectrometer’s detection efficiency of characteristic gamma rays with different energy from surface sources. The error thus introduced is acceptable in an in-situ measurement scenario in a nuclear emergency. This is because more accurate measurements are often achieved not by in-situ measurements but by sampling.

The detection efficiency of the spectrometer for the surface source should be the average of the point source detection efficiency in the detection area. According to the definition of the mean in probability theory,

8 $$ \overline{\varepsilon }_{E} = \frac{{\iint\nolimits_{D} {\varepsilon_{E} (r)d\sigma }}}{{\iint\nolimits_{D} {d\sigma }}} $$

where $\overline{\varepsilon }$_*E*_ represents detection efficiency of surface source with characteristic γ-ray with energy *E*; *ε*_*E*_(*r*) represents point source detection efficiency of characteristic γ-ray with energy *E* at a horizontal distance of r meters; *D* represents a circular area with the center of the detector’s vertical projection on the ground as the center and the effective contribution distance as the radius. Bringing the fitting Eq. (1) of point source detection efficiency into Eq. (8), we can get

9 $$ \overline{\varepsilon }_{E} = \frac{{2a[1 - (bd + 1)e^{ - bd} ]}}{{b^{2} \cdot d^{2} }} $$

where *a*, *b* represents the fitting coefficient of point source detection efficiency as a function of distance; *d* represents the effective contribution distance of the characteristic γ-ray with energy *E*, m; $\overline{\varepsilon }$_*E*_ represents the detection efficiency of the surface source with characteristic γ-ray with energy *E*. The characteristic γ-ray with different energies correspond to different adequate contribution distances, which are brought into the Eq. (10) for calculation, and the detection efficiency of the uniformly distributed surface sources can be obtained, as is shown in Table 4.

**Table 4:** Detection efficiency of uniformly distributed large area sources.

Radionuclide	Energy (keV)	Detection efficiency of surface source
^132^Te	228	1.34E−05
^131^I	364	9.12E−06
^133^I	530	6.64E−06
^137^Cs	662	5.48E−06
^60^Co	1173	3.47E−06
^60^Co	1332	3.12E−06
^124^Sb	1691	2.58E−06

**Figure 4:**
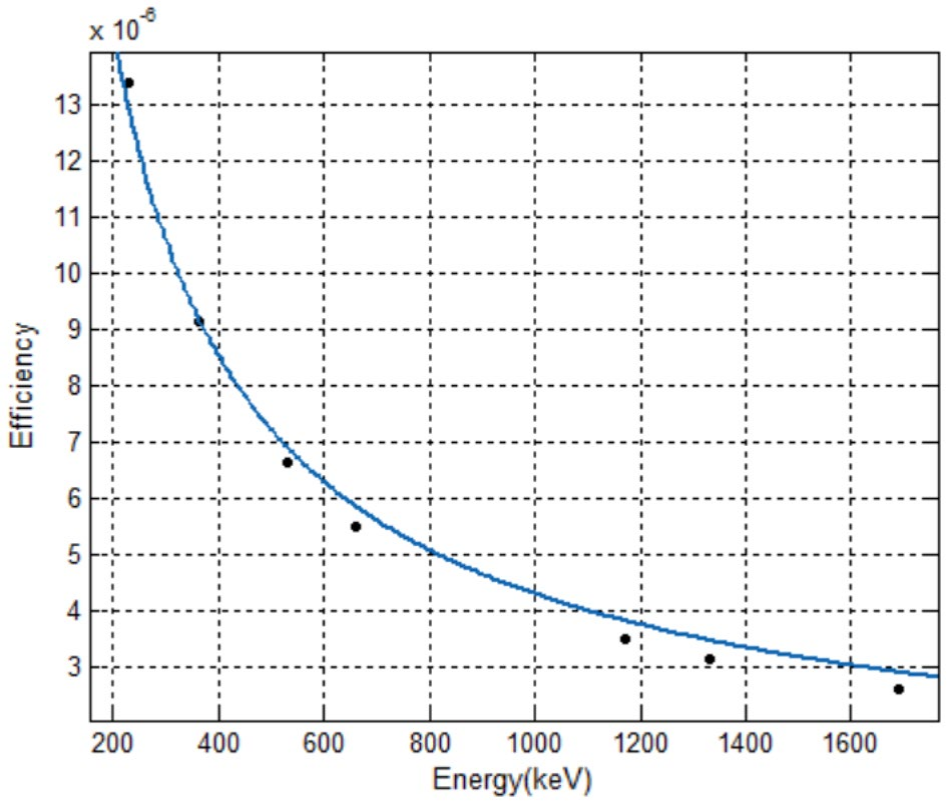
Fitting curve of HPGe γ spectrometer’s detection efficiency of radioactive surface source with characteristic γ-ray energy.

The detection efficiency and energy of the spectrometer for surface source are fitted by least squares, and the fitting equation is selected as follows,

10 $$ \overline{\varepsilon } = k_{1} \cdot E^{{k_{2} }} $$

where $\overline{\varepsilon }$ represents detection efficiency of surface source; *E* represents the energy of characteristic γ-ray, keV; *k*_1_, *k*_2_—fitting coefficient. The values of the fitted parameters are *k*_1_ = 0.001327, *k*_2_ =  − 0.8261, and the fitted regression coefficient *R*^2^ = 0.9992. The relationship between the spectrometer’s detection efficiency of uniformly distributed surface sources and the energy of characteristic γ-ray is shown in Fig. 4.

Observing the relationship between the detection efficiency and the energy, we can find that the detection efficiency of the surface source approximately follows the power function distribution and decreases monotonically with the increase of the energy. The greater the energy of γ-ray, the lower the detection efficiency in the effective contribution area is. It is consistent with the tendency of the detection efficiency of point sources to change with energy at the same position.

Due to the background count, the detection capabilities of different detection models are different. In the case of low activity, it is more difficult to determine whether the measured counts are due to pollutants or background. Only when the activity of a pollutant exceeds a certain threshold can it be judged as a pollutant.

The meaning of the lower detection limit is the limit at which the radioactivity can be detected by the detector, see Eq. (11)

11 $$ DL = (K_{\alpha } + K_{\beta } )\sqrt {n_{b} + n_{s} } , $$

where *K*_*α*_ represents the probability of false conclusions that there is radioactivity above the background in the sample and that there is no radioactivity above the background; *K*_*β*_ represents the probability of false conclusions that there is no radioactivity above the background in the sample to judge that there is radioactivity above the background; *n*_*b*_ represents background count rate, cps; *n*_*s*_ represents total count rate, cps. For low-level measurements, the total count rate can be approximately equal to the background count rate, that is

12 $$ n_{b} \approx n_{s} . $$

Let the probability *α* and *β* of both types of errors be 5%

13 $$ K_{\alpha } = K_{\beta } . $$

The *DL* can be expressed as the minimum detectable activity concentration (MDAC) ^21^, which is related to the parameters of the detection model. The calculation method for the MDAC is

14 $$ MDAC = \frac{{4.65\sqrt {tn_{b} } }}{tp\varepsilon SK}, $$

where *t* represents measurement time, s; *n*_*b*_ represents the background count rate of the nuclide’s region of interest, cps; *p* represents emissivity of γ-ray; *ε* represents detection efficiency of surface source in effective contribution area; *S* represents the area of effective contribution area, m^2^; *K* represents decay correction factor during measurement.

**Figure 5:**
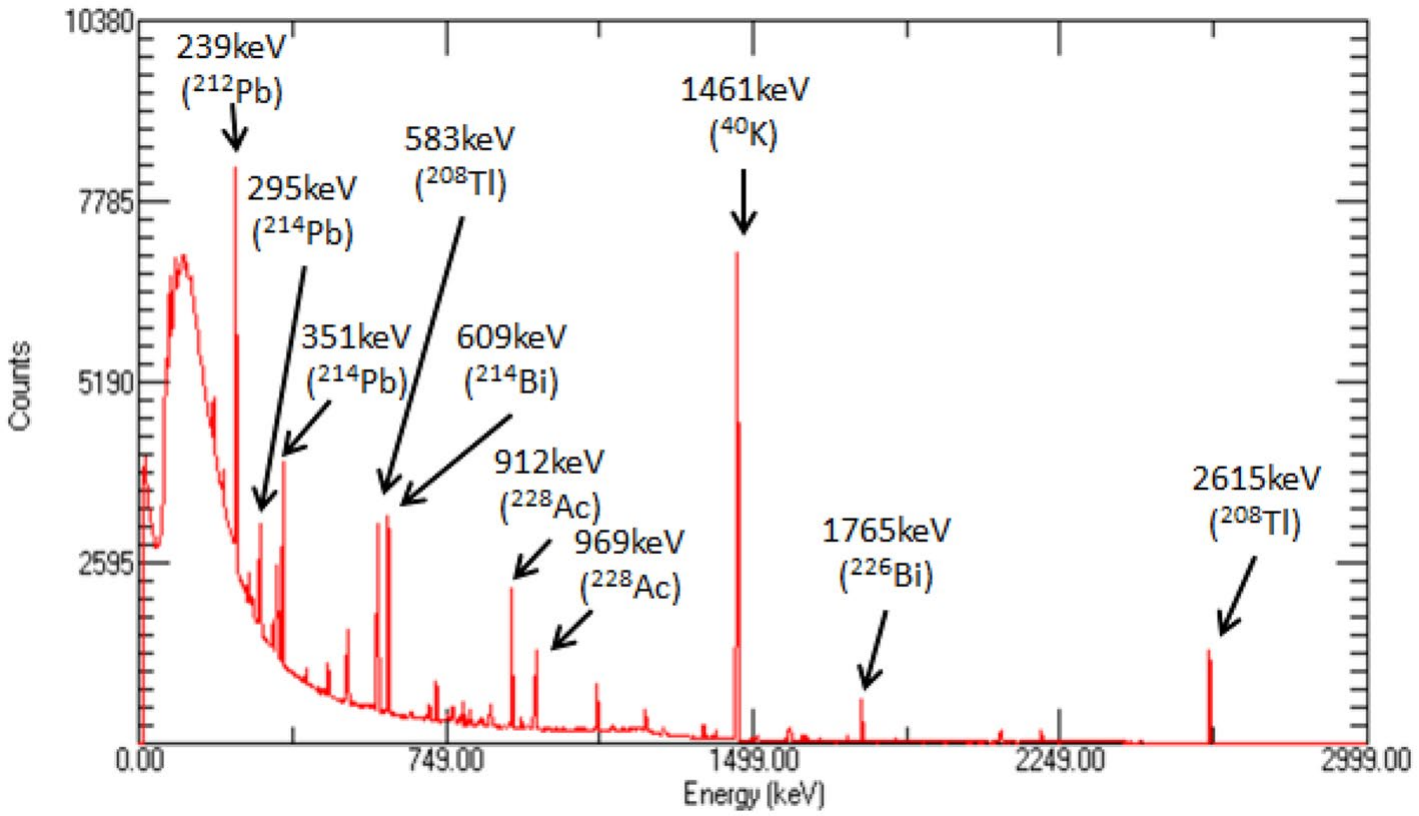
Background energy spectrum obtained from outdoor measurement at 15000 s. The characteristic γ-rays of various natural radioactive backgrounds are marked with arrows.

In order to obtain the background count rate as accurately as possible, the spectrometer is used to measure the outdoor background 15,000 s. The energy spectrum is shown in Fig. 5.

For characteristic γ-ray of different energy, the corresponding region of interest should be selected, and the background count within it calculated. Table 5 shows the count rate of the background in the region of interest of different energy γ-ray.

**Table 5:** Background measurement results of different energy γ-ray.

Radionuclide	Energy (keV)	Region of interest (keV)	Count	Count rate (cps)
^132^Te	228	[226.0, 230.0]	222,187	2.57
^131^I	364	[361.9, 366.1]	81,725	0.95
^133^I	530	[527.8, 532.2]	41,771	0.48
^137^Cs	662	[659.7, 664.3]	34,369	0.40
^60^Co	1173	[1170.3, 1175.7]	17,166	0.20
^60^Co	1332	[1329.2, 1334.8]	9358	0.11
^124^Sb	1691	[1687.9, 1694.1]	3445	0.04

In order to correct the decay of radionuclides during the measurement, according to the half-life of different nuclides, Eq. (3) was adopted to calculate the decay correction factors of different nuclides at different measurement times, as is shown in Table 6.

**Table 6:** Decay correction factors *K* for detection models.

Nuclide (energy/keV) (s)	^132^Te (228)	^131^I (364)	^133^I (530)	^137^Cs (662)	^60^Co (1173)	^60^Co (1332)	^124^Sb (1691)
100	1.000	1.000	1.000	1.000	1.000	1.000	1.000
1000	0.999	1.000	0.995	1.000	1.000	1.000	1.000
3600	0.996	0.998	0.984	1.000	1.000	1.000	1.000
7200	0.991	0.996	0.967	1.000	1.000	1.000	1.000

It can be seen that the decay of the radionuclide during the measurement has little effect on the measurement results. When measuring 7200 s for ^133^I with a half-life of 20.8 h, the minimum *K* is 0.967.

According to the calculation equation of the minimum detectable surface activity MDAC, the relationship between the value of MDAC and the types of radionuclides, the energy of γ-ray, and measurement time is shown in Table 7.

**Table 7:** Minimum detectable surface activity of the detection model (unit: Bq/m^2^).

Nuclide (energy/keV) (s)	^132^Te (228)	^131^I (364)	^133^I (530)	^137^Cs (662)	^60^Co (1173)	^60^Co (1332)	^124^Sb (1691)
100	6.43	5.89	5.28	5.62	5.19	4.08	5.99
1000	2.03	1.86	1.68	1.78	1.64	1.29	1.89
3600	1.08	0.98	0.89	0.94	0.86	0.68	1.00
7200	0.76	0.70	0.64	0.66	0.61	0.48	0.71

## Results

**Figure 6:**
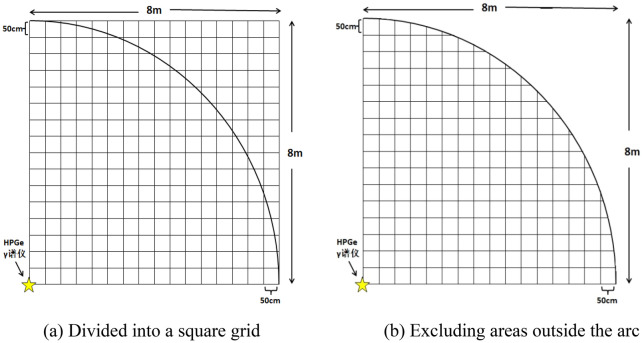
Simulation of uniformly distributed surface sources using the grid layout method. (**a**) shows the meshing principle, and the part enclosed by the arc in (**b**) is the area of interest.

In order to verify the rationality and validity of the simulation results, it is necessary to conduct experiments using a surface radioactive source. The effective contribution distance of the spectrometer to γ-rays with different energies is approximately 6 m. Under non-nuclear emergency conditions, it is difficult to measure the surface source of uniformly distributed artificial radionuclides with a radius greater than 6 m. Therefore, the grid point method is used to approximate the radioactive surface source. The schematic is shown in Fig. 6.

As shown in Fig. 6, a square grid with a side length of 8 m and an interval of 0.5 m is established, and the spectrometer is set up at the apex of the square, as shown in Fig. 6a. At the same time, with the spectrometer position as the center of the circle and 8 m as the radius to make the arc, only the area inside the arc is considered, as shown in Fig. 6b. If the same radioactive source is sequentially placed at the intersection of the grids at this time, and the measurement time is the same, it can be approximately equivalent to measuring a uniformly distributed quarter-surface radioactive surface source with a radius of 8 m. Its activity is the surface activity of the micro-elements represented by the radioactive point source, and the simulated measurement time is 1/215 of the actual measurement time. According to the symmetry, the energy spectrum of the spectrometer for a uniformly distributed surface source with a radius of 8 m can be calculated. The activities of the standard radioactive point sources used are shown in Table 8.

**Table 8:** Radioactivity of standard point sources.

Radionuclide	Activity (Bq)	Relative uncertainty (%, k = 2)
^137^Cs	24,380	1.7
^60^Co	24,403	1.7

**Figure 7:**
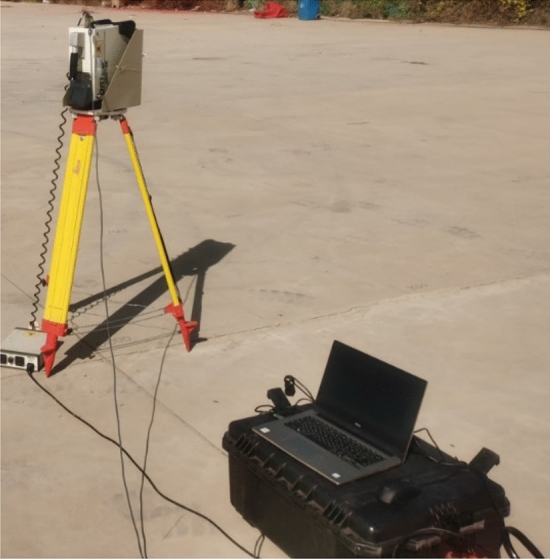
The experimental site was chosen to be in a flat and open area, and the HPGe γ spectrometer was set up at the height of 1 m from the ground and connected to a computer. Place the radioactive sources in sequence according to the grid division method described in Fig. 6 to complete the experiment.

**Figure 8:**
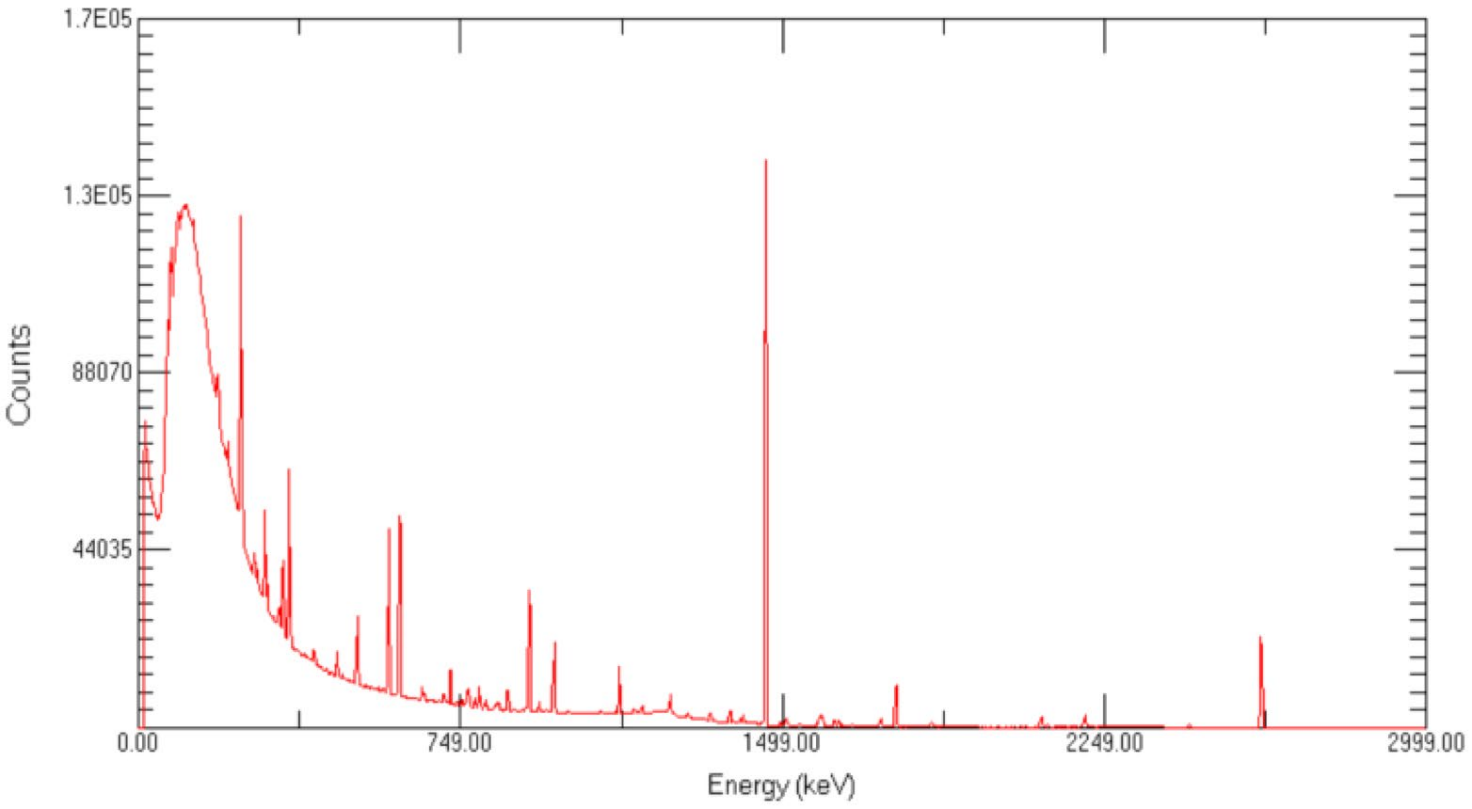
Energy spectrum of a uniformly distributed surface source experiment using the grid method.

There are 215 intersections and vertices, and each point measures the 1200 s, and the total measurement time is 258,000 s. The experimental site is shown in Fig. 7, and the energy spectrum is shown in Fig. 8.

The experimental results are shown in Table 9.

**Table 9:** Grid source experiment results.

Radionuclide	Energy (keV)	Net count	Uncertainty range	Time (s)	Net count rate (cps)
^137^Cs	662	15,380	± 635	258,000	5.96E−02
^60^Co	1173	12,680	± 657	258,000	4.91E−02
^60^Co	1332	11,397	± 457	258,000	4.42E−02

Since the detector is placed at the apex of the grid, the experimentally measured part is a quarter of the actual detection object. In order to transform the experimental results into the measurement results of the uniformly distributed radioactive surface source by the spectrometer, according to the symmetry, the net count rate, surface activity, and other values need to be expanded four times.

The surface activity of a uniformly distributed surface source simulated by the grid method can be calculated by the Eq. (15)

15 $$ A_{S} = \frac{{215A_{p} }}{{\pi R^{2} }} \times 4, $$

where *A*_*S*_ represents the surface activity of uniformly distributed surface source, Bq/m^2^; *A*_*p*_ represents the activity of the point source used in the experiment, Bq; *R* represents the radius of uniformly distributed area source simulated by grid method, m, here is 8 m. The calculation of the net count rate of the simulated surface source is shown in the Eq. (16)

16 $$ n_{S\_tr} = 215n_{S\_or} \times 4, $$

where *n*_*S_tr*_ represents the net count rate of simulated surface source, cps; *n*_*S_or*_ represents the actual net count rate measured, cps.

According to the measurement results of the net count rate, the detection efficiency of surface source, and effective contribution distance, the surface activity can be calculated by the Eq. (17)

17 $$ A_{S\_MC} = \frac{{n_{S\_tr} }}{{\varepsilon_{S\_MC} \cdot P_{r} \cdot \pi d_{MC}^{2} }}, $$

where *A*_*S_MC*_ represents surface activity derived from Monte Carlo simulation of point source detection efficiency, Bq/m^2^; *ε*_*S_MC*_ represents detection efficiency of surface source; *d*_*MC*_ represents effective contribution distance derived by Monte Carlo simulation of point source detection efficiency, m; *P*_*r*_ represents emissivity of characteristic γ-ray.

Compare the derived activity with the activity calculated by the Eq. (17), and the validity of the calibration method is tested. Substituting the effective contribution distance and surface source detection efficiency in Table 1 into the calculation, the comparison results between the theoretical and integral calculations of surface source activity are shown in Table 10.

**Table 10:** Comparison of surface source experiment results with Monte Carlo simulation detection efficiency .

Radionuclide	Energy (keV)	Net count rate of simulated source (cps)	Surface activity of simulated source (Bq/m^2^)	Surface activity of integral calculation (Bq/m^2^)	Relative error (%)
^137^Cs	662	51.3	1.04E+05	9.83E+04	−5.4
^60^Co	1173	45.6	1.04E+05	1.14E+05	9.6
^60^Co	1332	44.7	1.04E+05	1.19E+05	14.6

It can be seen that the relative error of the measurement of uniformly distributed radioactive surface sources using the numerical integration method is relatively small, all of which do not exceed 14.6%. The results of surface source detection efficiency obtained by this method are relatively accurate.

## Conclusion

In this paper, a method for rapid surface activity measurement of radioactive surface sources settled on the surface under nuclear emergency conditions is studied. Innovatively introduce the numerical integration method into the process of calibration of surface source detection efficiency. The area of ​​numerical integration is a circular area enclosed within the effective contribution distance. Since the detection efficiency of point sources is simulated by Monte Carlo method, and the detection efficiency of surface sources is calculated by the numerical integration, this method is reproducible. The calculation of point source detection efficiency uses the Monte Carlo simulation method, which has the advantage that: (1) the results are obtained quickly; (2) no natural radioactive source is required for calibration. The disadvantages are: (1) the spectrometer needs to be characterized before use because the HPGe γ spectrometer will change its dead layer thickness and size the hole with the use of time, which will cause errors in the simulation results; (2) There are some differences between the simulation model and the real situation, such as a spectrometer tripod, air humidity, etc., which will cause certain errors.

After the point source detection efficiency is calculated, the horizontal distance corresponding to 95% of the maximum contribution rate extending outward from the projection point of the detector is defined as the effective contribution distance, and the numerical integration method is used to calculate the detection efficiency of the surface source. Finally, the grid point method is used to simulate the radioactive surface source for experiments. The maximum relative error between the theoretical calculation and the experiment is 14.6%, which verifies the effectiveness of this method.

Since radioactive contamination may exist not only on the surface but also in seawater under nuclear emergency conditions, an effective in situ measurement means is needed. Since the attenuation coefficient of γ-rays in seawater is different from that in air, and the spatial distribution of radioactive substances in seawater is unknown, the method cannot be directly applied to measurements in seawater, and further relevant studies are worthwhile in the future.

## Data Availability

The datasets generated and analysed during the current study are available in the Mendeley Data repository, https://doi.org/10.17632/h59f2wnc5x.1.
